# Development of machine-vision system for gap inspection of muskmelon grafted seedlings

**DOI:** 10.1371/journal.pone.0189732

**Published:** 2017-12-21

**Authors:** Siyao Liu, Zuochang Xing, Zifan Wang, Subo Tian, Falalu Rabiu Jahun

**Affiliations:** 1 College of Engineering, Shenyang Agricultural University, Shenyang, China; 2 Department of Agricultural Engineering, Bayero University, Kano, Nigeria; Huazhong Agriculture University, CHINA

## Abstract

Grafting robots have been developed in the world, but some auxiliary works such as gap-inspecting for grafted seedlings still need to be done by human. An machine-vision system of gap inspection for grafted muskmelon seedlings was developed in this study. The image acquiring system consists of a CCD camera, a lens and a front white lighting source. The image of inspected gap was processed and analyzed by software of HALCON 12.0. The recognition algorithm for the system is based on principle of deformable template matching. A template should be created from an image of qualified grafted seedling gap. Then the gap image of the grafted seedling will be compared with the created template to determine their matching degree. Based on the similarity between the gap image of grafted seedling and the template, the matching degree will be 0 to 1. The less similar for the grafted seedling gap with the template the smaller of matching degree. Thirdly, the gap will be output as qualified or unqualified. If the matching degree of grafted seedling gap and the template is less than 0.58, or there is no match is found, the gap will be judged as unqualified; otherwise the gap will be qualified. Finally, 100 muskmelon seedlings were grafted and inspected to test the gap inspection system. Results showed that the gap inspection machine-vision system could recognize the gap qualification correctly as 98% of human vision. And the inspection speed of this system can reach 15 seedlings·min^-1^. The gap inspection process in grafting can be fully automated with this developed machine-vision system, and the gap inspection system will be a key step of a fully-automatic grafting robots.

## Introduction

Grafting is a technique that graft branches or buds from one plant with appropriate parts of another plant that has a strong affinity. A branch or bud named scion to be cultivated is supposed to be combined with a strong plant named rootstock, so that the scion can grow, blossom and bear. Grafting technology can solve the continuous cropping obstacles, increase yield and fruit quality [1]. Grafting robot technology has recently been developed. In many countries, some semi-automatic grafting robots for cucumber, tomato and watermelon have been developed and commercialized [2]. The working efficiency of semi-automatic grafting robots is three times faster than the efficiency of traditional manual grafting [3]. So far, most automatic grafting machine’s speed can reach 600 seedlings/h [4–6]. However, since the machines still require manual work then they can only achieve a semi-automatic [7]. The quality of the grafted gap must be inspected so that transplanting survival rate can be improved. However, present semi-automated grafting robots are still need manual gap quality inspection, the gap inspection method need to be improved.

Machine-vision has been applied in many research fields, breeding industry, fishery, agriculture and so on. In recent years, pattern recognition study of machine-vision has been applied in various fields For example in inspecting quality of grafted seedlings [8], acquiring parameters of seedling such as, cotyledon node information, seedling stem diameter and seedling stem bending angle [9]. Image processing algorithms is the most important in machine-vision [10–14]. The main algorithms include that, color space conversion algorithms, gray-scale transformation, image binaryzation processing algorithms[15–18]. Although some algorithms can distinguish the characteristics of inspected object in detail, the calculation process of them are still too complex, hence not suitable for inspecting the gap of grafted seedling like muskmelon. Furthermore, because of the variety of seedlings, some algorithm is very simple and usually give inaccurate results. For this reason, it is important to design a simple and practical gap inspection machine-vision system for muskmelon grafting machine.

The aim of this study was to develop an automatic gap inspection machine-vision system for muskmelon grafted seedling. Objectives of the study are: to select appropriate hardware (camera, lens, and lighting source) for image acquisition, to develop algorithm of gap inspection machine-vision system and to conduct gap quality inspection test of muskmelon grafted seedling.

## Materials and methods

### Image acquisition

**Fig 1:**
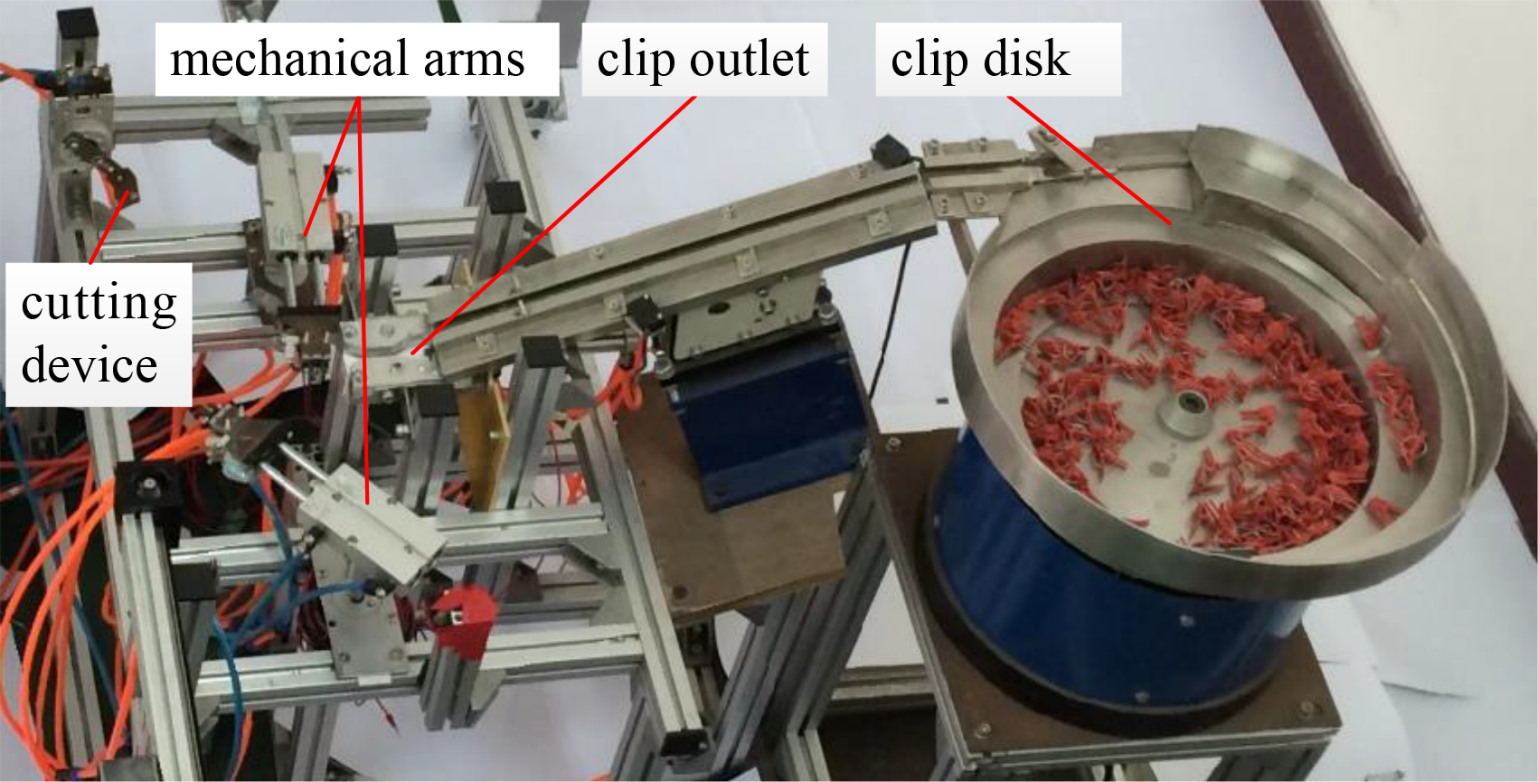
Main structure of automated grafting machine.

The seedling grafting was carried out using an automated grafting machine developed by our research team [19]. The main structure of the grafting machine is as shown in Fig 1. The machine’s grafting process is as follows: Firstly rootstock (pumpkin) and scion (muskmelon) were held by two respective mechanical arms and then sent them to the cutting device where they were cut to the same appropriate angle. The ready rootstock and scion were later aligned, put together and clamped using a clip. Finally, the grafted seedling was prepared for the image acquisition process.

### Camera selection

The hardware structures of the image acquisition system include: camera, lens and light source were selected separately. The selected camera is MER-125-30UC modelwhich has high resolution and suitable for acquiring shapes of very tiny objects such as muskmelon seedlings. Therefore, a complete and reliable images of the grafted seedlings can easily be acquired within a short distance.

The colored camera is equipped with 1/3” Sony ICX445 CCD sensor chip. The sensor chip size is 4.8mm×3.6mm with resolution of 1292×964 pixels. Specific performance parameters of the camera are shown in Table 1.

**Table 1:** CCD camera MER-125-30UC performance parameters table.

Type	MER-125-30UC
Resolution	1292(H)×964(V)
Frame	30 fps
Type of sensor chip	1/3” Sony ICX445 CCD
Size of pixel	3.75μm × 3.75μm
Data interface	Mini USB 2.0
Size	29×29×29 mm
Weight	41 g

The camera can also connect directly with some image processing software such as HALCON to set appropriate exposure and other parameters to acquire and process images.

### Lens selection

**Fig 2:**
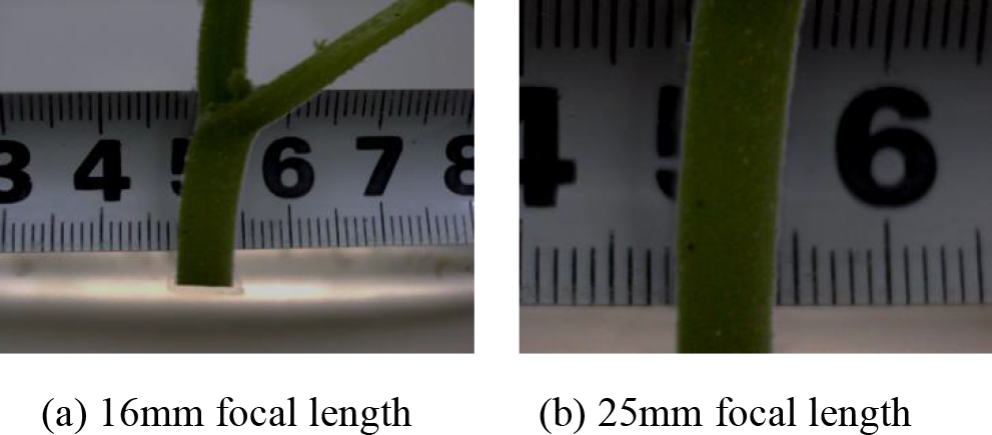
Images of seedling stem from lenses with different focal lengths.

In selecting appropriate lens for grafted seedling gap inspection, the characteristics of grafting clip and the clamped stem portion were considered. In this way, the main characteristics of the grafting clip and clamped stem should be very clear. Two lenses of different focal lengths (f) 16 and 25mm were tested. Two images of the seedling stem with same object distance were acquired with each lens, to get the best image as shown in Fig 2. The seedling stem images acquired with 25mm focal length lens were found to be clearer, and more conspicuous. The images obtained (f = 25mm) also had fewer background information hence, more suitable for inspecting the grafted seedling gap. Consequently, lens with focal length, f = 25mm was selected for image acquisition.

### Light source selection

**Fig 3:**
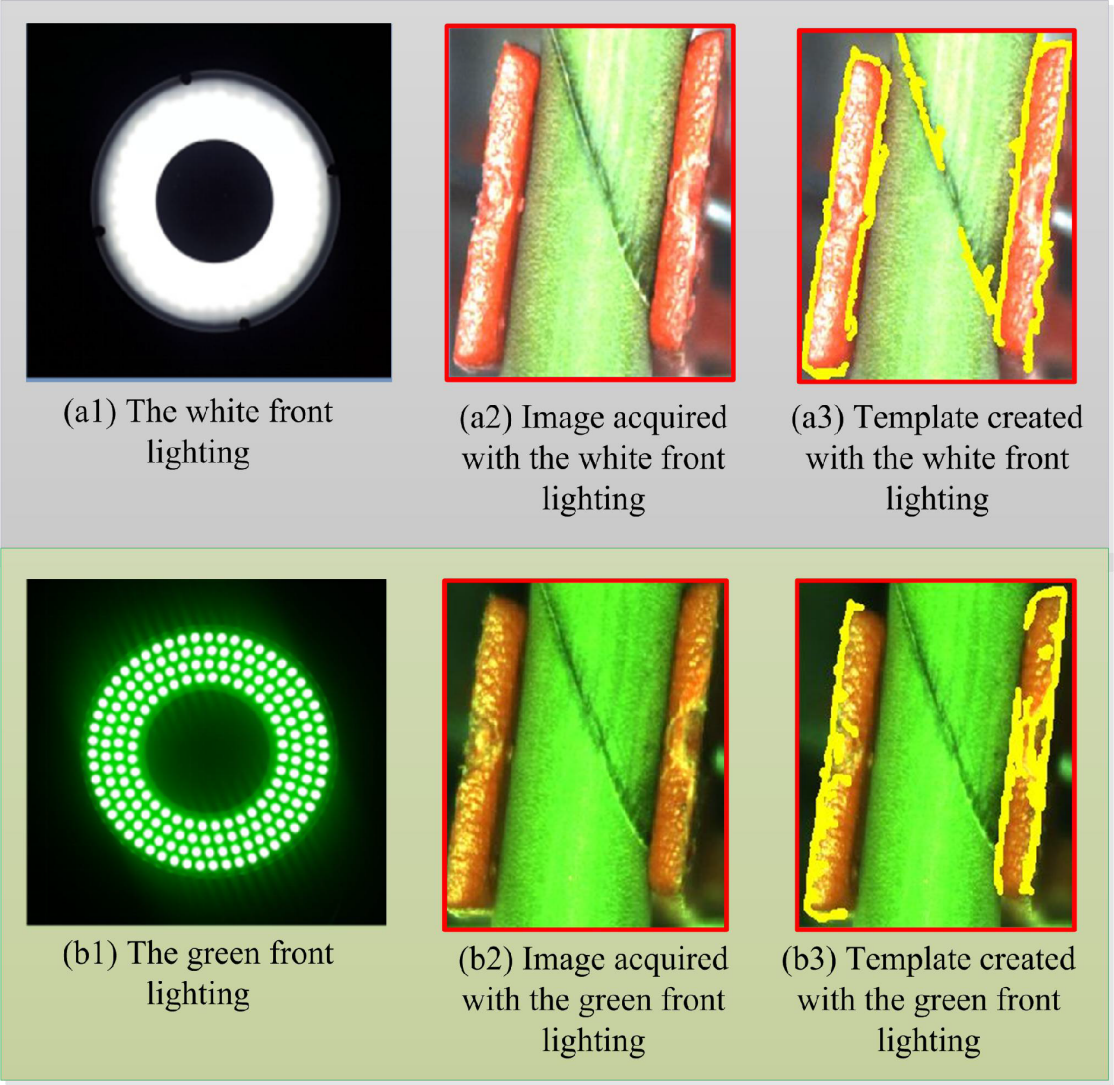
The illumination schemes for the experiment.

Light source plays an important role in the machine-vision system. The image acquired with natural light usually has too much noise hence it will not give proper illumination, and white light or green light may enhance the contrast between grafting gap and background, while lighting source with other color can’t make the grafting gap clearer. Therefore, two lighting schemes:, the white LED ring front lighting source, and green LED ring front lighting source were selected and tested. The lighting sources and their respective images acquired together with the templates created are shown in Fig 3. In the figure, (a1) and (b1) are the white and the green lighting source, while (a2) and (b2) are the images acquired with the white and green lighting sources respectively. Also, (a3) and (b3) depict the templates created from the images acquired with the white and green lighting sources. It was clearly observed that, for the same seedling, a much clearer template was created with the white front lighting and the intensity of natural light will not affect the images acquired, but an incomplete and unclear template was obtained with the green front lighting. Therefore, the white front lighting was selected for acquiring image.

**Fig 4:**
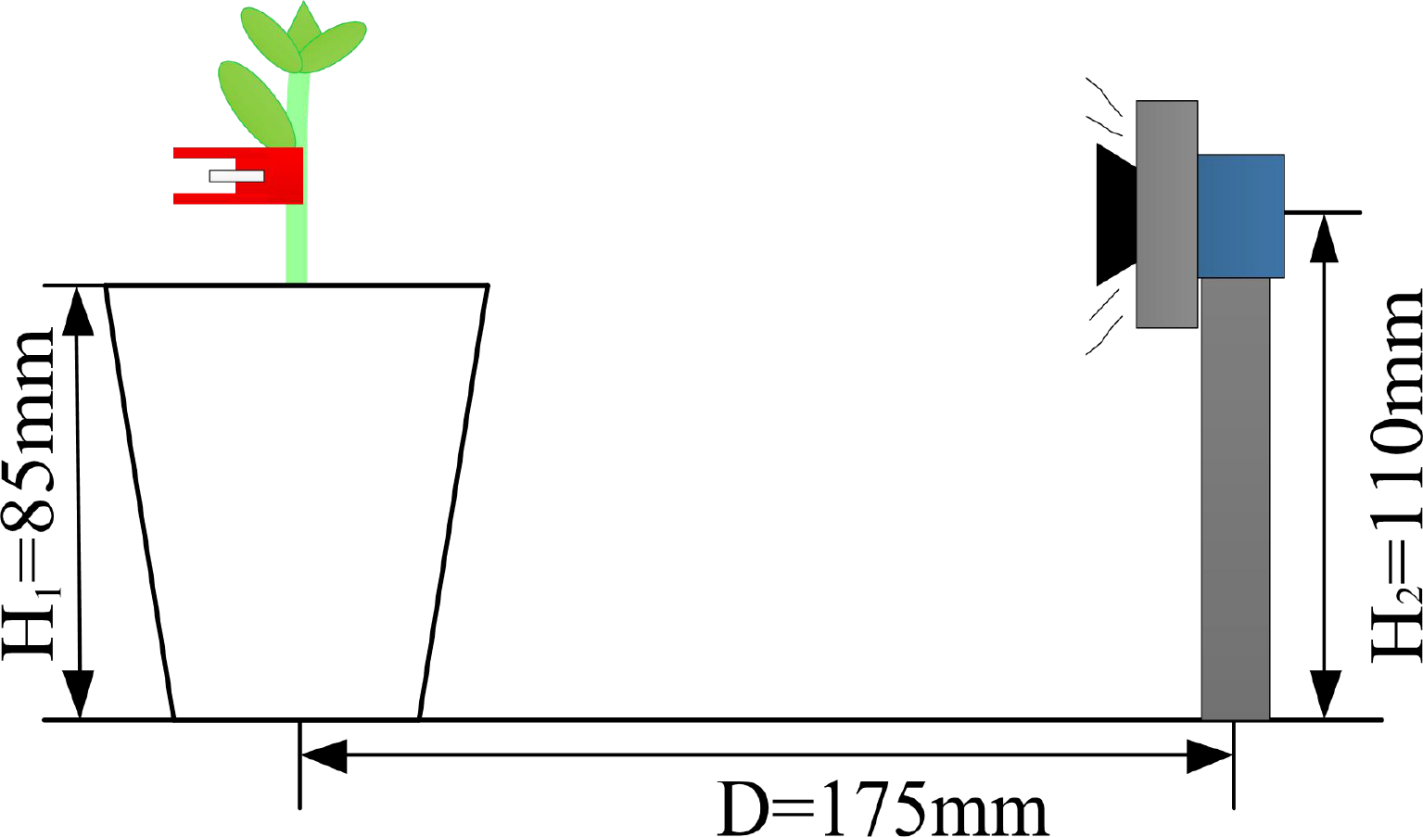
Image acquisition layout.

**Fig 5:**
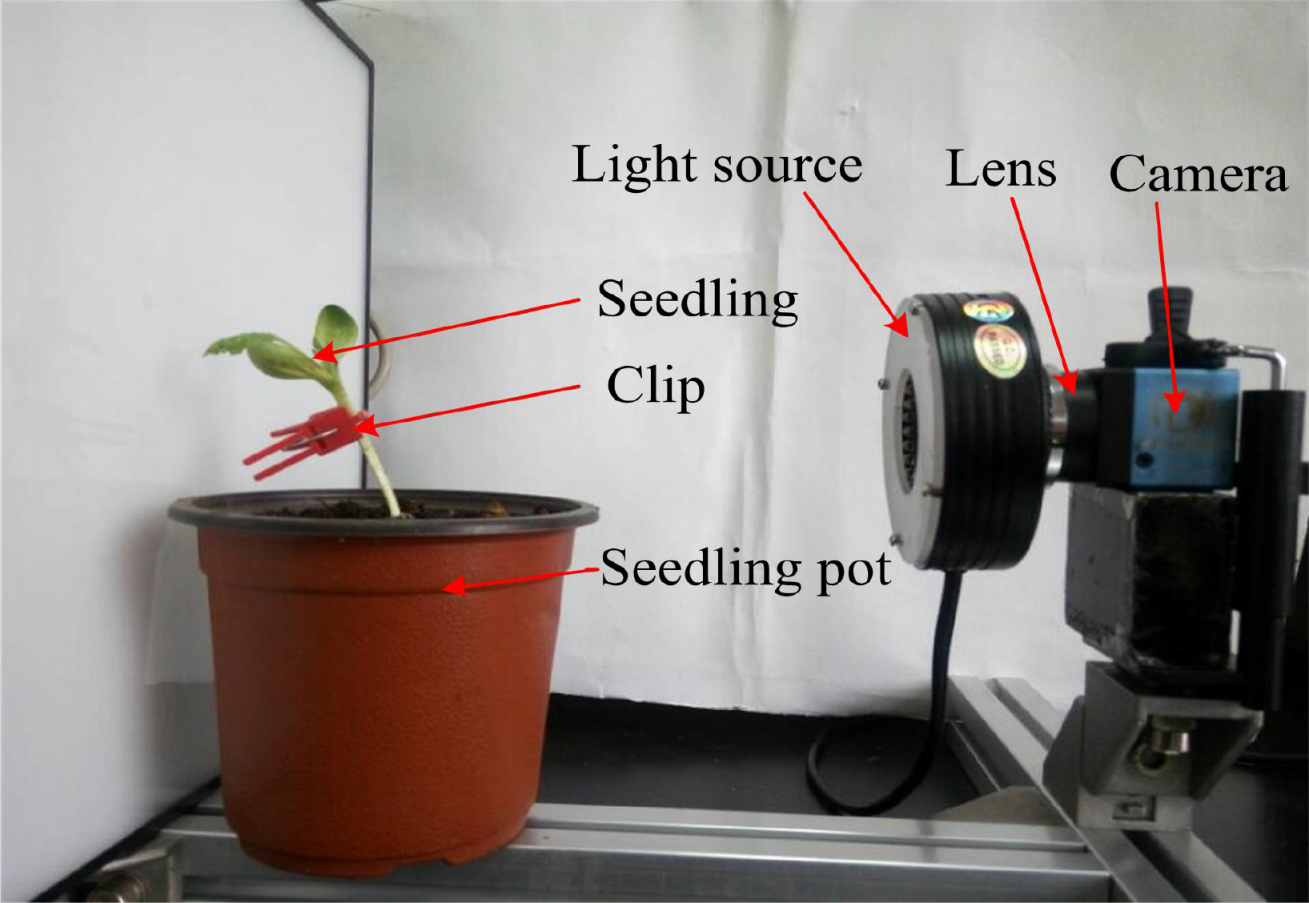
Image acquisition structure.

The grafted seedlings are not vertical in general and the images are acquired difficulty. The height of seedling pot is 85mm, the distance between the grafted gap and the pot rim is 20mm to 30mm, thus the height of the camera was set as 110mm. The working distance of selected lens with focal length 25mm is over 50mm. In order to acquire whole image of grafted gap, the center of seedling pot and the camera was set in a horizontal line, and the distance between them was set as 175mm which was tested as best. Finally, the hardware of machine vision system was set up. The hardware layout and structure are shown in Figs 4 and 5. The ring front lighting is set on the lens and and the distance between the center of seedling pot and the camera (D) was set as 175mm. The height of seedling pot (H1) is 85mm, distance between center of camera and the working plane (H2) was set as 110mm.

### Gap inspection of grafted seedlings

#### Grafted gap inspection standard

The grafted seedlings were inspected by physical observation and prepared to be inspected of qualification.Two necessary conditions must be satisfied for a qualified grafted seedling thus: (1) Rootstock and scion must be grafted completely and gap fitted well. (2) The seedling must be held tightly by the grafting clip.

**Fig 6:**
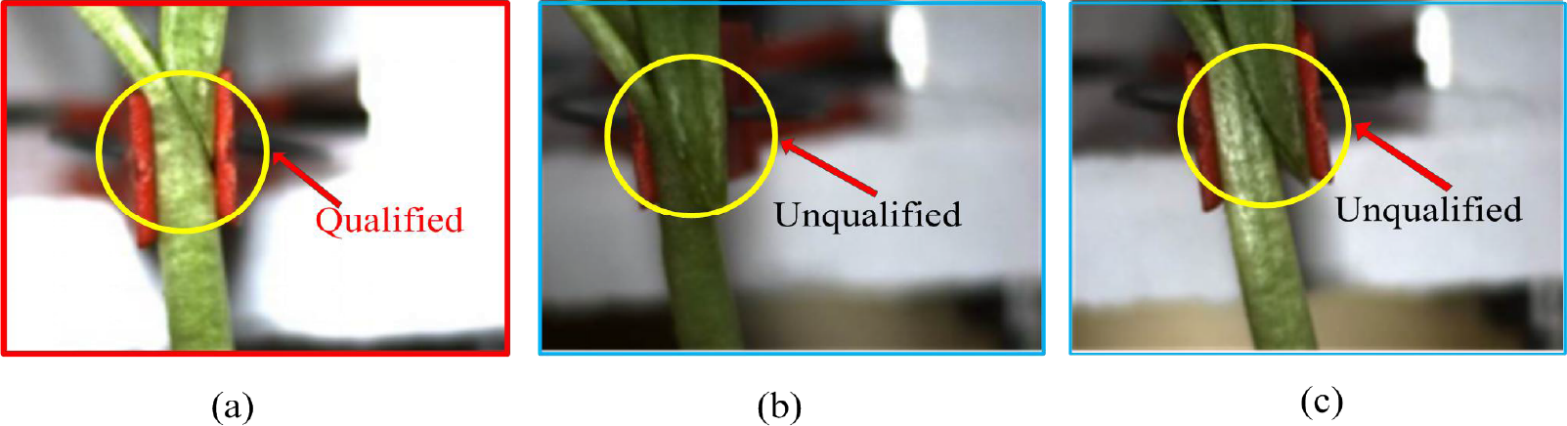
Three possible results of the grafted gap.

There are mainly three possible results are usually obtained (Fig 6) as follows: (a) Rootstock and scion are grafted completely, the gap is well fitted and seedling held tightly by the grafting clip. (b) Rootstock and scion are grafted completely, but the seeding is not held tightly. (c) Rootstock and scion are grafted incompletely, the grafted parts are not fitted well. Consequently, result in (a) is regarded as the qualified case, while results (b), (c) and any other conditions are considered as unqualified.

#### Template matching technique

HALCON 12.0 software was used, the system is a professional image processing software developed by MVtec. The software integrated development environment and flexible architecture makes it convenient for all kinds of automatic detection.

Due to the different shapes of grafted gap, traditional image processing methods could not be able to directly recognize grafted gap. Therefore, a better and more reliable method of template matching has been adopted by many researchers in different fields, and this method is also used in this study to recognize the grafted gap[20–23]. Template matching method can calculate the similarity between the template characteristics and the image to be inspected. In this study the template matching based on deformable template technique was selected to recognize the grafted gap, since it is sensitive to deformation. The theory of template matching is as following: The size of template T is supposed as M×N, and the size of target image S is L×W(L>M, W>N). Template matching means overlapping the template T onto the target image, and the similarity between the template and sub-image S_i,j_ can be measured based on the following equations:

$$D(i,j)=\sum_{m=1}^{M}\sum_{n=1}^{N}\left[S_{i,j}(m,n)-T(m,n\right){]}^{2}$$ (1)

Expansion formula (1) shown as following:

$$D(i,j)=\sum_{m=1}^{M}\sum_{n=1}^{N}{\left[T(m,n)\right]}^{2}+\sum_{m=1}^{M}\sum_{n=1}^{N}[S_{i,j}(m,n){]}^{2}-2\sum_{m=1}^{M}\sum_{n=1}^{N}S_{i,j}(m,n)\times T(m,n)$$ (2)

In the Eq (2), the first term is the energy of the template which will not change with the template position (i,j); the second term is the energy of the sub-image which will change a little with the template position; and the last term of the equation means the cross correlation energy between the template and the sub-image, and this term will reach its maximum value when the template and the sub-image are totally matched. So the similarity between the template and sub-image can be calculated with the following equation:

$$R(i,j)=\frac{\sum_{m=1}^{M}\sum_{n=1}^{N}S_{i,j}(m,n)\times T(m,n)}{\sum_{m=1}^{M}\sum_{n=1}^{N}{\left[S_{i,j}(m,n)\right]}^{2}}$$ (3)

And the Eq(3) can be normalized as Eq(4):

$$R(i,j)=\frac{\sum_{m=1}^{M}\sum_{n=1}^{N}S_{i,j}(m,n)\times T(m,n)}{\sqrt{\sum_{m=1}^{M}\sum_{n=1}^{N}{\left[S_{i,j}(m,n)\right]}^{2}}\sqrt{\sum_{m=1}^{M}\sum_{n=1}^{N}{\left[T(m,n)\right]}^{2}}}$$ (4)

And based on the Cauchy-Schwarz inequality, the similarity R(i,j) is a number between 0 to 1 (in this study “score”), signify the similarity between the template characteristics and the found characteristics.

**Fig 7:**
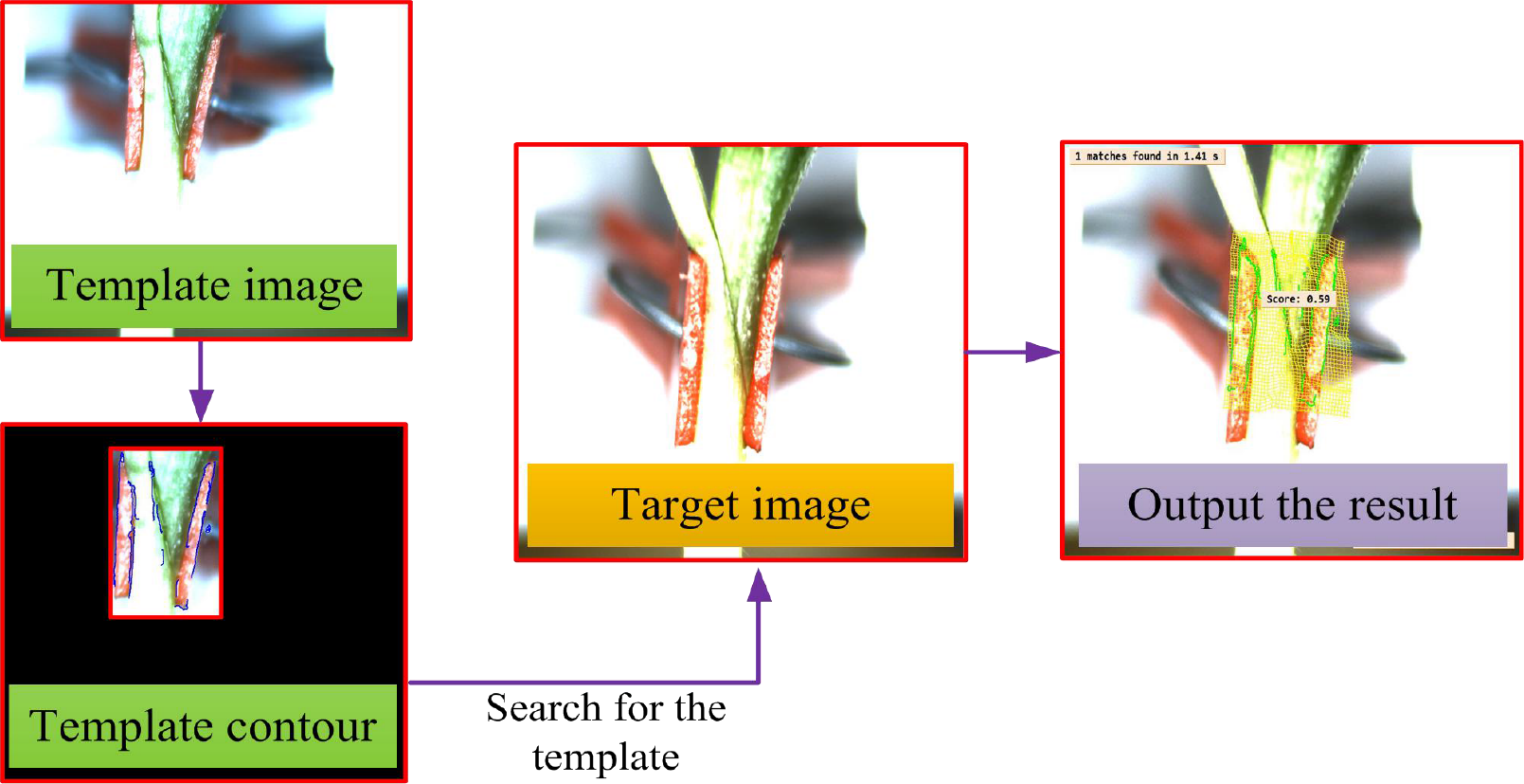
Flowchart of template matching process.

The technique basically consist of template creation, searching for the template from the target images, and outputting the inspecting result. The flowchart of the template matching process is as shown in Fig 7 below.

**Fig 8:**
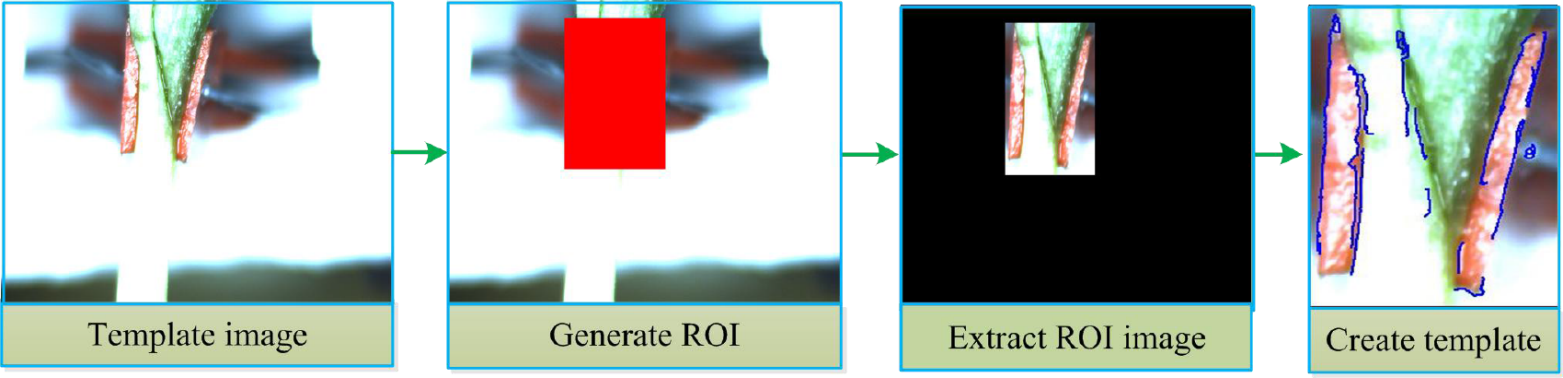
Flowchart of template creation.

**Template matching steps. 1) Template creation:** During the process of template creation, important parameters were first defined, these include, pyramid level, starting angle and rotation range of the template, periodic angle of template rotation and the contrast between the background and target object. The best clear image was selected as the template image, by generating a ROI (Region of Interest). The selected ROI is a region filled with the typical characteristics (the gap and the grafting clip holding it) and was separated out from background. The template was then created based on the characteristics in the ROI image with the given parameters. The template contours include the grafting clip and the grafted gap. Fig 8 shows pictorial flowchart of template creation process.

**Fig 9:**
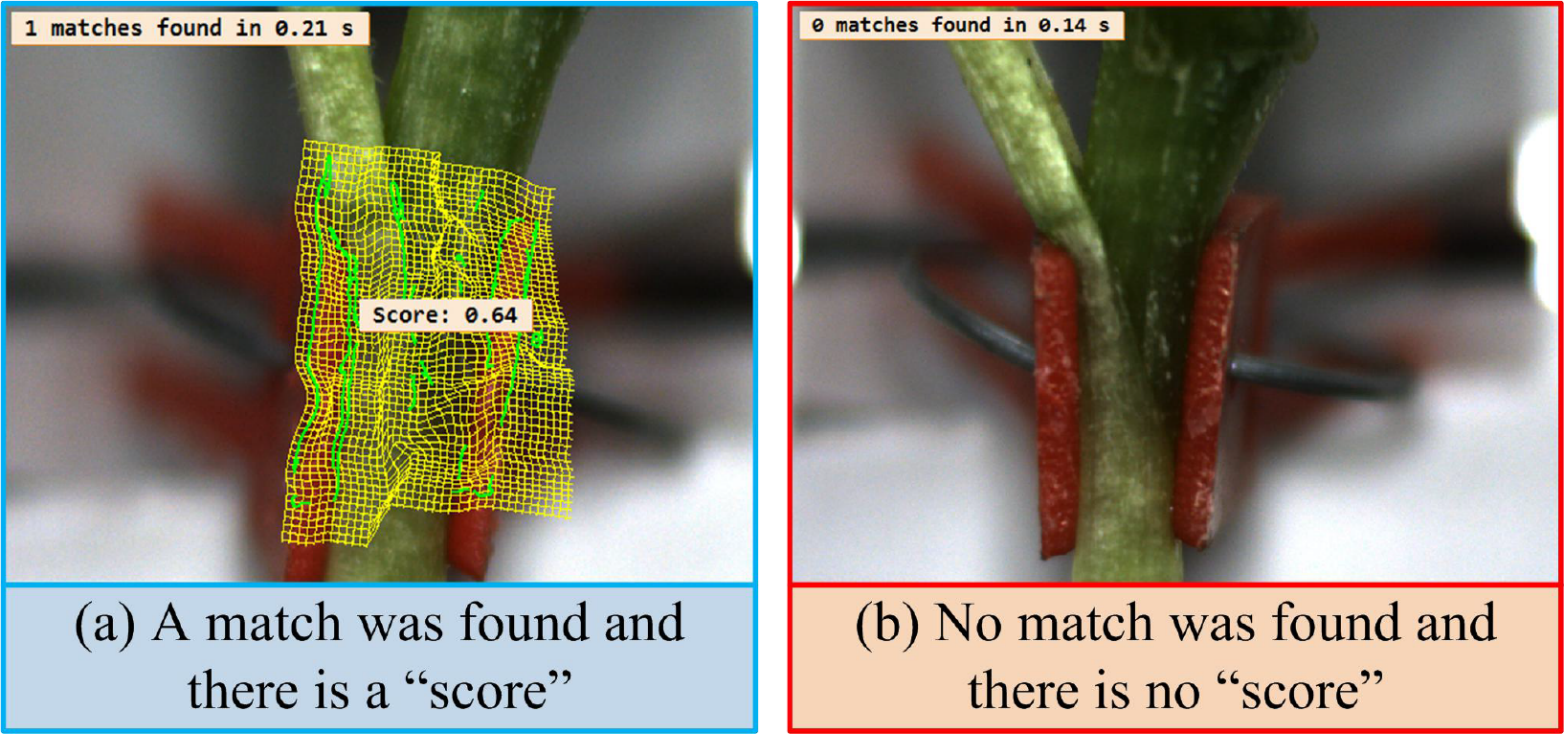
Possible result of researching for template in the target image.

**2) Searching for the created template:** The deformable matching can be used to detect an object that is distorted by a local deformation. The purpose of searching for the template is to find the best match of a deformable template in the target image. In order to find the best match in the target images, some important defined parameters were optimized for the template searching thus: The angle of start and rotation range of the template, number of template searching, the largest overlap ratio, and greediness degree. Before searching of the created template from the target image, the program firstly rectify the shape change of contours characteristics of the target image, then search for the template in the target image according to the given parameters. After searching of the template in the target image, if a match is found, a number between 0 and 1 (score) will be output, as shown in the Fig 9A. However, if no match is found, there will not be a “score”, as shown in the Fig 9B.

**3) Outputting the inspection result:** After searching for the template among many target images, some results obtained for the qualified and unqualified grafted gaps are shown in Table 2 below. These result shows that scores of the qualified grafted gaps are greater than the unqualified ones. This means that qualified grafted gaps have more similar contours with that of template contour are more. Through the data comparison it was observed that the minimum score value of qualified gaps(0.58) is always greater than the maximum value of the unqualified gaps. Consequently, a threshold score value of 0.58 was established. Therefore, if the score of a match is less than 0.58 the gap will be considered unqualified, else it is unqualified.

**Table 2:** Sample results of searching for the template in the target grafted gap images.

Experimental run	1	2	3	4	5	6	7	8	9	10
Qualified grafted gaps scores	0.87	0.68	0.76	0.95	0.85	0.73	0.62	0.92	0.59	0.94
Unqualified grafted gaps scores	—	0.26	0.43	—	0.31	—	—	0.48	0.27	0.33

“—” means no match was founded

#### Developing the grafted gap inspection algorithm

**Fig 10:**
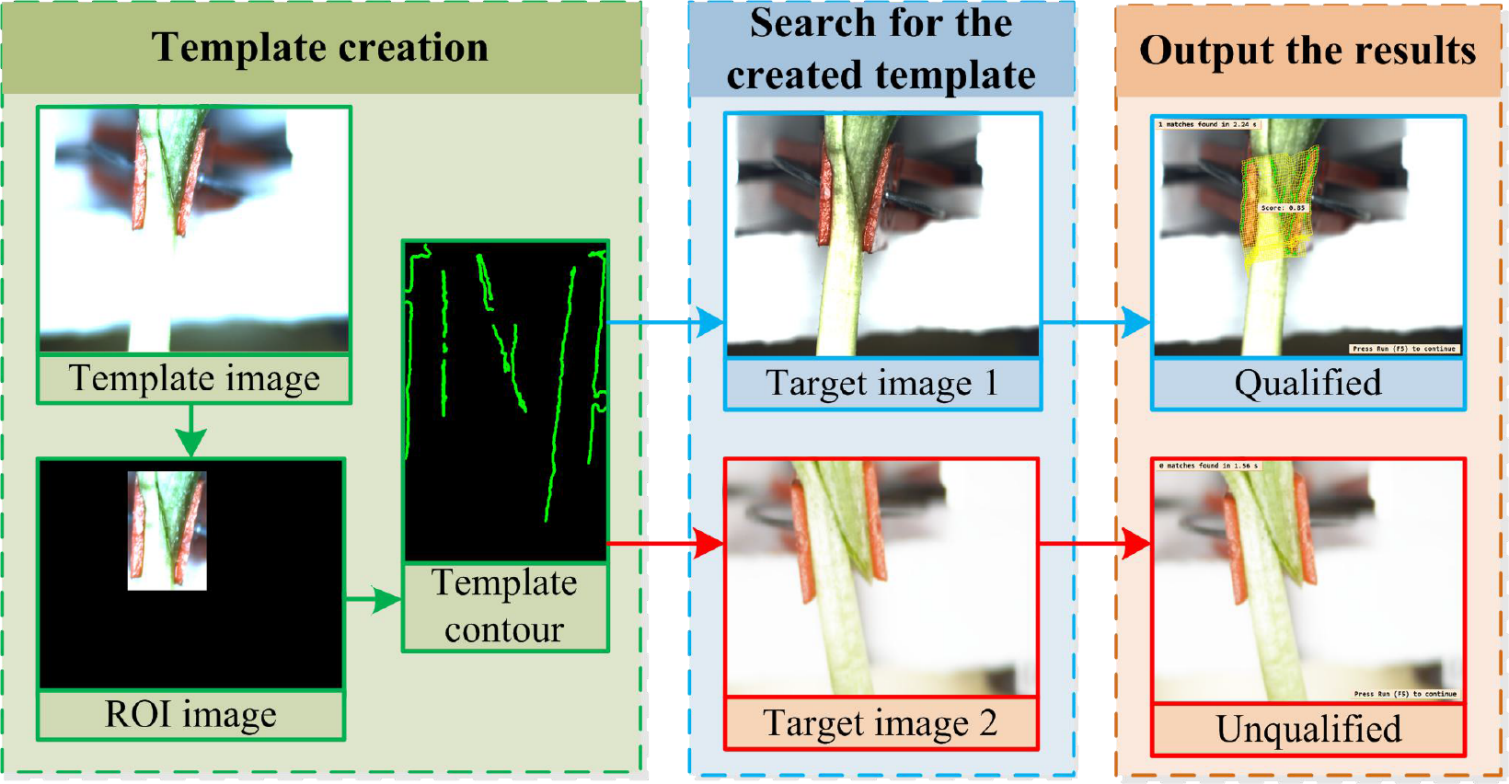
The output of the program algorithm displayed on the interface.

The developed algorithm for the template matching process is as following: (1) Read the template image;(2) Create the template;(3) Search for the template in target image;(4) Inspect the grafted gap quality with searching result(if “score” is equal to or greater than 0.58, the grafted gap will be judged as qualified, else if “score” is less than 0.58 or there is no match found in the target image, the grafted gap will be judged as unqualified).The output of the program algorithm as displayed on the interface is also shown in Fig 10.

More so, the main grafted gap inspection algorithm programmed with HALCON are as follow:

1. Read an image using the operator “read_image()”;
2. Generate a ROI using the operator “gen_rectangle1()”;
3. Create a deformable template using the operator “create_local_deformable_model()”;
4. Get the characteristic contour of the template using the operator “get_deformable_model_contours()”;
5. Search for the template in the target image using the operator “find_local_deformable_model()”.

## Results and discussion

The developed machine-vision system was tested to inspect the gaps qualities of 100 muskmelon grafted seedlings. The inspection results showed there are 8 unqualified seedlings, however in actual observation, there are only 6 unqualified seedlings. That means there were 2 qualified seedlings are inspected by the system as unqualified ones. Therefore, the failure rate of the gap inspection machine-vision system is 2%.

**Fig 11:**
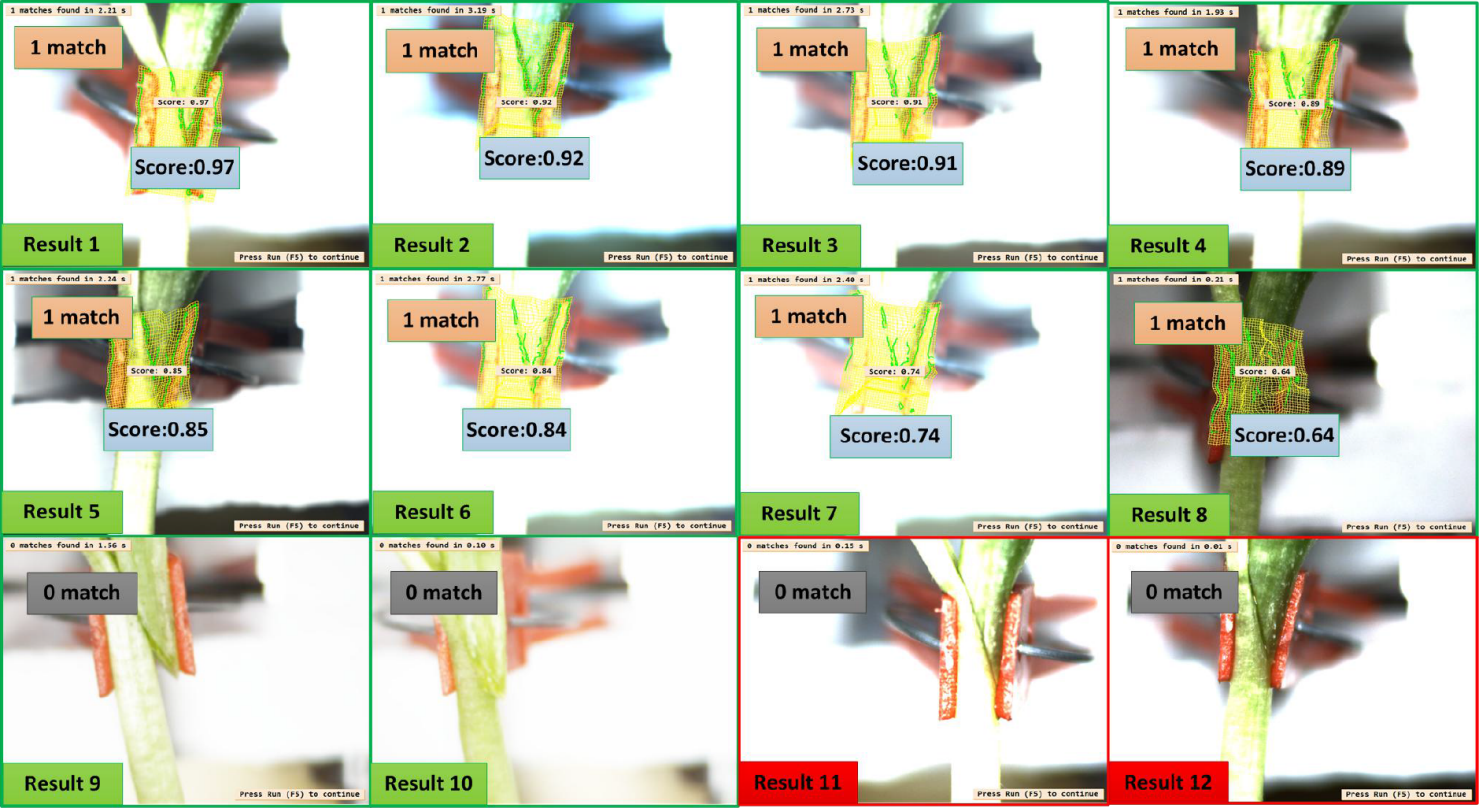
Template matching results.

12 samples were selected among these 100 tested grafted seedlings showed in Fig 11. Result 1 to 8 depict the output of the inspected seedlings as qualified, and the results are the same with the actual. The scores obtained range from 0.97 to 0.64, however it should be noted that a score doesn’t mean the success degree of the seedling, rather it only gives the degree of similarities between the seedling and the template. Similarly, results 9 and 10 show the output of two seedlings that inspected as unqualified, and were actually found physically to be unqualified. Their rootstocks and scions were seen not grafted completely. In addition, the last two results (results 11 and12) show the fail inspection result but actually, these two seedlings are qualified ones, even though the inspection output shows as unqualified grafted seedlings. The reason for this failure could be due to large bending angles of the seedlings, because it will cause the distance between the center of the camera and the gap too far or too near, and unclear grafted gap will be acquired. Also, if the seedling bending angle is too large, the template matching algorithm could not find the template in the target image, then the inspection will be a failure. In some condition the presence of soil and water on the seedling will destroy the contour of the gap image, and cause the inspection to fail.

It was observed that average time taken to inspect a seedling using the machine-vision system is less than 4 seconds (the inspection speed of this system can reach 15 seedlings·min^-1^.), based on experiment results, which can fulfill the speed demand of the automatically grafting process. However, it takes much longer time to inspect the grafted seedling manually in the actual production. Therefore the application of machine-vision system for gap quality inspection can improve the efficiency and automation degree of grafting process, and can give an important meaning in grafting robots development.

## Conclusion

In this study, a machine-vision system for gap inspection of muskmelon grafted seedlings based on template matching was developed. The hardware of the system was selected and set up, and the gap inspection algorithm was developed based on the deformable template matching. The test indicates that the inspection speed of this grafted gap inspection system can reach 15 seedlings·min^-1,^ and, the inspection success rate is 98%, which can fulfill the demand of the automation of grafting robots with great application value. The high success rate machine-vision gap inspection system provides a theoretical basis for further research of automatic grafting robots.

## Supporting information

S1 File Detection results of 100 tested grafted seedlings. (XLSX)
